# Platelet-to-lymphocyte ratio and telomere length in older adults: An inverted U-shaped nonlinear relationship: A nationwide cohort study

**DOI:** 10.1097/MD.0000000000044188

**Published:** 2025-09-12

**Authors:** Qinqin Liu, Yue Gu, Haixia Huang, Jiawei Ren, Yang Liu, Delong Wang

**Affiliations:** a Department of Geriatrics, Jiangwan Hospital of Hongkou District, Shanghai, PR China; b Department of Geriatrics, Yantai Affiliated Hospital of Binzhou Medical University, Yantai, PR China; c Department of Rehabilitation Medicine, Jiangwan Hospital of Hongkou District, Shanghai, PR China; d Medical Affairs Department, Jiangwan Hospital of Hongkou District, Shanghai, PR China; e Department of Neurology, Minhang Hospital, Fudan University, Shanghai, PR China.

**Keywords:** aging, inflammation, nonlinear relationship, platelet-to-lymphocyte ratio, telomere length

## Abstract

This study aimed to investigate the association between the platelet-to-lymphocyte ratio (PLR) Log and telomere length in older adults, focusing on the potential nonlinear relationship within a nationwide cohort. Data were obtained from the National Health and Nutrition Examination Survey 1999 to 2000 and 2001 to 2002 cycles, including 2660 participants aged 60 years and older. PLR Log was calculated as the log-transformed value of PLR, which was further analyzed as both a continuous variable and in quartiles. Mean telomere length (TeloMean) was measured using quantitative PCR. Linear regression, trend tests, smooth curve fitting, and segmented regression were employed to evaluate the relationship between PLR Log and TeloMean, adjusting for potential confounders. Subgroup analyses were conducted to assess variations in associations across age, sex, and other variables. An inverted U-shaped nonlinear relationship was identified between PLR Log and TeloMean. Trend analysis demonstrated a significant trend across quartiles of PLR Log in both the minimally adjusted (*P* for trend = .021) and fully adjusted models (*P* for trend = .048). Threshold effect analysis identified a breakpoint at PLR Log = 5.564, where TeloMean significantly increased with PLR Log when below this threshold (β1 = 0.034, 95% CI: 0.012–0.057, *P* = .003), but decreased when PLR Log exceeded this threshold (β2 = −0.115, 95% CI: −0.221 to −0.009, *P* = .033). This association was more pronounced in participants aged 66 to 74 years (*P* for interaction = .005). This study provides new insights into the relationship between systemic inflammation and telomere dynamics in older adults. The observed inverted U-shaped relationship suggests that moderate levels of inflammation may help maintain telomere integrity, possibly through improved immune regulation, while excessive inflammation may accelerate telomere attrition due to increased oxidative stress and impaired DNA repair mechanisms. These findings highlight PLR Log as a potential inflammatory biomarker for aging-related telomere dynamics and emphasize the need for further longitudinal studies to validate these associations.

## 1. Introduction

Telomeres are protective structures located at the ends of chromosomes that gradually shorten with each cell division, serving as important biomarkers of aging and age-related diseases.^[1]^ Telomere shortening has been linked not only to natural aging processes but also to cardiovascular diseases, diabetes, and neurodegenerative conditions. Chronic systemic inflammation has been identified as a key driver of accelerated telomere shortening, likely due to increased oxidative stress and direct DNA damage.^[2–4]^ Blackburn first established the relationship between telomere length, cellular aging, and inflammation.^[5]^ Liu et al^[6]^ further demonstrated that chronic inflammation could accelerate telomere shortening through multiple pathways. Understanding these interactions is therefore crucial to fully comprehending the aging process.

The platelet-to-lymphocyte ratio (PLR) is a simple marker for monitoring systemic inflammation, combining platelet and lymphocyte counts to reflect the body’s pro-inflammatory state and immune regulatory function.^[7]^ PLR has been associated with the development and prognosis of several chronic diseases, including cardiovascular conditions, metabolic disorders, and cancer.^[8–10]^ An elevated PLR typically indicates an active pro-inflammatory state and declining immune function, a profile characteristic of “inflammaging.” Tang et al proposed that PLR not only assesses systemic inflammation levels but also serves as a potential predictor of age-related diseases and the aging process itself.^[11]^

Though several studies have linked PLR with various diseases, little research has explored its relationship with telomere length, particularly among older adults. Pro-inflammatory cytokines and immune imbalance may accelerate telomere shortening through multiple mechanisms. Bednarski found that reactive oxygen species released by neutrophils can directly damage DNA, while a decrease in lymphocyte count may impair DNA repair mechanisms,^[12]^ suggesting PLR may exhibit an irregular, nonlinear relationship with telomere length.

This study aims to assess the relationship between PLR and telomere length among older adults aged 60 years or older using data from the National Health and Nutrition Examination Survey (NHANES). We hypothesize that an inverted U-shaped nonlinear association exists, where moderate elevations in PLR are associated with longer telomeres, while excessively high PLR levels may lead to telomere shortening. Our findings may provide new insights into the complex mechanisms of inflammation and telomere dynamics in older populations.

## 2. Materials and methods

### 2.1. Data source

The data used in this study were obtained from the NHANES, conducted by the National Center for Health Statistics (NCHS). NHANES is a nationally representative cross-sectional survey designed to assess the nutritional status and potential health risks of U.S. population. To ensure national representation, NHANES uses a complex, stratified, multistage probability sampling design. Participants were interviewed in their homes to collect socioeconomic and health-related information, and they underwent laboratory tests and physical examinations at mobile examination centers.

The NHANES protocol was approved by the NCHS Research Ethics Review Board. All participants provided written informed consent, and for those under 16 years of age, consent was obtained from their parents or legal guardians. Detailed information about the NHANES study design and data is publicly available on the official website (www.cdc.gov/nchs/nhanes/).

### 2.2. Study population

**Figure 1.**
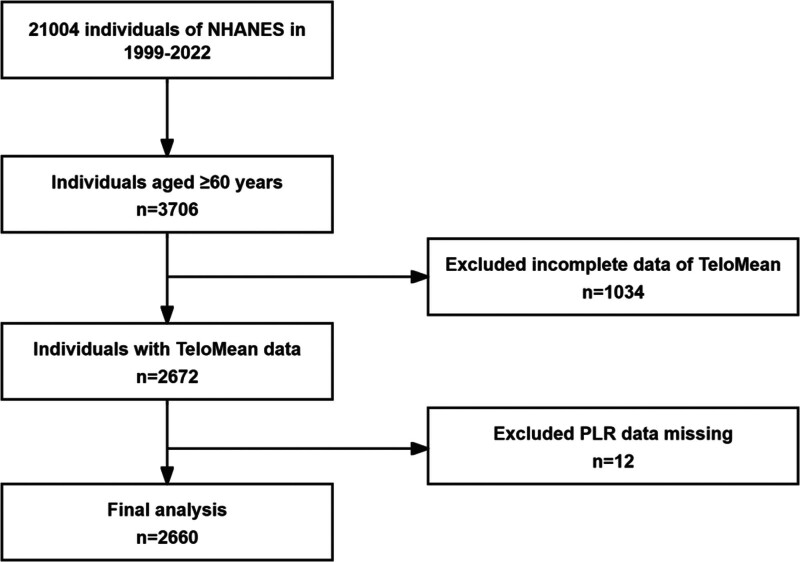
Flow chart of study participants.

This study analyzed data from 2 NHANES cycles (1999–2000 and 2001–2002), as telomere length was measured only during these periods. The analysis included all participants with complete telomere and PLR data. Initially, there were 21,004 participants, but after excluding those younger than 60 years (n = 17,298), those without telomere data (n = 1034), and individuals missing PLR information (n = 12), a total of 2660 participants met the study criteria and were included in the final analysis (Fig. 1).

### 2.3. Definition of PLR

In this study, PLR was used as the exposure variable. PLR was calculated by dividing the platelet count by the lymphocyte count using the formula: PLR = platelet count/lymphocyte count. PLR was analyzed as both a continuous variable and grouped according to quartiles.

### 2.4. Telomere assessment

Telomere length was assessed using blood samples from NHANES participants aged 60 and older during the 2 study cycles. The relative telomere length was measured by quantitative polymerase chain reaction, comparing telomere sequence copy number (T) to a single-copy reference gene (S), expressed as the T/S ratio. Each sample was measured 3 times on different days, resulting in 6 data points per sample. Each plate contained 96 control wells, including 8 control DNA samples. If >8 control wells were invalid or >4 control DNA samples exceeded 2.5 standard deviations (SDs) from the mean, the results from that plate were excluded. Outliers for individual samples were also removed. The mean T/S ratio (mean telomere length [TeloMean]) and SD were used to evaluate telomere length. In this study, TeloMean was analyzed as the outcome variable.

### 2.5. Assessment of covariates of interest

Based on a review of the literature, potential confounders were controlled in this study. These included sex (male/female), age (years), race/ethnicity (Mexican American/other Hispanic/non-Hispanic White/non-Hispanic Black/other race [including multiracial]), marital status (widowed/divorced/separated/never married/living with partner), education level (<9th grade/9–11th grade/high school graduate/GED or equivalent/some college or associate’ s degree/college graduate or above), smoking history (smoked ≥ 100 cigarettes/smoked < 100 cigarettes), alcohol consumption (≥2 drinks per day/<2 drinks per day), diabetes (yes/no), and hypertension (yes/no). These factors were considered as they may influence the association between PLR and TeloMean. Diabetes and hypertension were self-reported in the questionnaire.^[13]^ Detailed methods for measuring these variables can be accessed on www.cdc.gov/nchs/nhanes/.

### 2.6. Statistical analysis

Statistical analyses were performed using R software version 4.2.0 (R Foundation for Statistical Computing, Vienna, Austria). Descriptive statistics were conducted to present the baseline characteristics across quartiles of PLR. Continuous variables were expressed as mean ± SD, and categorical variables were presented as frequencies and percentages. Differences between groups were assessed using one-way analysis of variance or the Kruskal–Wallis test for continuous variables, while categorical variables were analyzed using the Chi-square test. To explore the association between PLR and telomere length, linear regression models were used to evaluate the relationship between PLR quartiles and telomere length, with stepwise adjustments for confounding factors including sex, age, race, marital status, education level, smoking history, alcohol consumption, diabetes, hypertension, and body mass index (BMI). Additionally, a trend test was performed to assess the potential dose–response relationship between PLR quartiles and telomere length. PLR was also analyzed as a continuous variable, and to address its skewed distribution, log transformation (PLR Log) was applied. Furthermore, we conducted smooth curve fitting and segmented regression analysis to assess potential nonlinear relationships and identify the inflection point for PLR. Subgroup analyses were conducted based on age, sex, and other characteristics to examine variations in the effect within different subgroups. All statistical tests were 2-sided, and a *P*-value of <.05 was considered statistically significant.

### 2.7. Ethics statement

The study involving human participants was approved by the NCHS Ethics Review Board. All participants provided written informed consent, indicating their voluntary participation in the NHANES study.

## 3. Results

### 3.1. Baseline characteristics of study participants

The characteristics of the study participants, stratified by PLR quartiles, are presented in Table 1. A total of 2660 participants were included, with a mean age of 71.45 ± 7.85 years, and a nearly equal sex distribution. Statistically significant differences were observed across PLR quartiles for variables such as sex, race, marital status, education level, smoking status, diabetes, hypertension, BMI, neutrophil count, platelet count, lymphocyte count, Systemic Immune-inflammation Index (SII), and TeloMean (*P* < .05). Compared to the PLR Q1 group, TeloMean progressively increased in the Q2 and Q3 groups (Q1: 0.885 ± 0.232, Q2: 0.899 ± 0.218, *P* = .007), but slightly decreased in the Q4 group (Q4: 0.899 ± 0.215). Additionally, the SII was significantly higher in the PLR Q4 group compared to other groups (Q1: 347.93 ± 134.77, Q4: 1055.70 ± 753.30, *P* < .001).

**Table 1:** Baseline characteristics of study participants by the PLR quartile.

Characteristic	PLR levels				
	Quartile 1 (n = 664)	Quartile 2 (n = 666)	Quartile 3 (n = 662)	Quartile 4 (n = 668)	*P*-value
Age (years)	70.992 ± 7.794	71.326 ± 7.936	70.597 ± 7.633	72.895 ± 7.997	<.001
Sex (%)					.838
Male	341 (51.355%)	329 (49.399%)	341 (51.511%)	344 (51.497%)	
Female	323 (48.645%)	337 (50.601%)	321 (48.489%)	324 (48.503%)	
Race (%)					.002
Mexican American	155 (23.343%)	146 (21.922%)	125 (18.882%)	126 (18.862%)	
Other Hispanic	40 (6.024%)	18 (2.703%)	29 (4.381%)	20 (2.994%)	
Non-Hispanic White	349 (52.560%)	378 (56.757%)	400 (60.423%)	426 (63.772%)	
Non-Hispanic Black	102 (15.361%)	111 (16.667%)	94 (14.199%)	87 (13.024%)	
Other race [including multiracial]	18 (2.711%)	13 (1.952%)	14 (2.115%)	9 (1.347%)	
Education level (%)					<.001
<9th grade	215 (32.380%)	179 (26.877%)	149 (22.508%)	148 (22.156%)	
9–11th grade	111 (16.717%)	132 (19.820%)	110 (16.616%)	110 (16.467%)	
High school graduate/GED or equivalent	138 (20.783%)	159 (23.874%)	159 (24.018%)	159 (23.802%)	
Some college or AA degree	114 (17.169%)	115 (17.267%)	134 (20.242%)	127 (19.012%)	
College graduate or above	86 (12.952%)	81 (12.162%)	110 (16.616%)	124 (18.563%)	
Marital status (%)					.028
Married	404 (60.843%)	433 (65.015%)	439 (66.314%)	402 (60.180%)	
Widowed	152 (22.892%)	143 (21.471%)	139 (20.997%)	188 (28.144%)	
Divorced	62 (9.337%)	55 (8.258%)	51 (7.704%)	38 (5.689%)	
Separated	17 (2.560%)	12 (1.802%)	10 (1.511%)	9 (1.347%)	
Never married	20 (3.012%)	14 (2.102%)	17 (2.568%)	26 (3.892%)	
Living with partner	9 (1.355%)	9 (1.351%)	6 (0.906%)	5 (0.749%)	
Smoking (%)					.359
≥100 cigarettes in life	371 (55.873%)	352 (52.853%)	338 (51.057%)	352 (52.695%)	
<100 cigarettes in life	293 (44.127%)	314 (47.147%)	324 (48.943%)	316 (47.305%)	
Alcohol (%)					.993
<2 drinks/day	518 (78.012%)	519 (77.928%)	513 (77.492%)	522 (78.144%)	
≥2 drinks/day	146 (21.988%)	147 (22.072%)	149 (22.508%)	146 (21.856%)	
Diabetes (%)					.002
Yes	141 (21.235%)	130 (19.520%)	99 (14.955%)	99 (14.820%)	
No	523 (78.765%)	536 (80.480%)	563 (85.045%)	569 (85.180%)	
Hypertension (%)					.37
Yes	339 (51.054%)	360 (54.054%)	326 (49.245%)	345 (51.647%)	
No	325 (48.946%)	306 (45.946%)	336 (50.755%)	323 (48.353%)	
Body mass index (kg/m^2^)	29.004 ± 5.389	28.632 ± 5.091	28.028 ± 5.201	27.311 ± 4.981	<.001
Neutrophils count (10^3^/μL)	4.194 ± 1.406	4.141 ± 1.333	4.145 ± 1.483	4.428 ± 1.781	.04
Platelet count (10^3^/μL)	218.047 ± 56.314	243.848 ± 56.317	261.266 ± 58.779	296.323 ± 87.586	<.001
Lymphocyte count (10^3^/μL)	2.739 ± 1.106	2.092 ± 0.502	1.771 ± 0.411	1.341 ± 0.386	<.001
SII	347.925 ± 134.768	484.811 ± 161.153	614.845 ± 227.837	1055.695 ± 753.301	<.001
TeloMean	0.885 ± 0.232	0.899 ± 0.218	0.917 ± 0.211	0.899 ± 0.215	.007

PLR = platelet-to-lymphocyte ratio, SII = Systemic Immune-inflammation Index, TeloMean = mean telomere length.

### 3.2. Association between PLR Log and TeloMean

The association between PLR Log and TeloMean is shown in Table 2. The study revealed a positive correlation between PLR Log and TeloMean, particularly pronounced in the Q3 group. In the unadjusted model, TeloMean was significantly greater in the Q3 group compared to the Q1 group (β = 0.032, 95% CI: 0.008–0.056, *P* = .008), and this trend persisted in the adjusted models. In the minimally adjusted model (adjusted for sex, age, and race), TeloMean in the Q3 group remained significantly higher than in the Q1 group (β = 0.028, 95% CI: 0.005–0.051, *P* = .016). Even in the fully adjusted model (further adjusting for education level, marital status, smoking status, alcohol consumption, diabetes, hypertension, and BMI), the Q3 group still showed a significant increase in TeloMean compared to the Q1 group (β = 0.025, 95% CI: 0.002–0.049, *P* = .030).

**Table 2:** Association between PLR Log and TeloMean.

Exposure	Crude model	Minimally adjusted model	Fully adjusted model
	Model 1	Model 2	Model 3
PLR Log Q1	Reference	Reference	Reference
PLR Log Q2	0.014 (−0.010, 0.037) 0.249	0.014 (−0.009, 0.037) 0.232	0.013 (−0.010, 0.036) 0.210
PLR Log Q3	0.032 (0.008, 0.056) 0.008	0.028 (0.005, 0.051) 0.016	0.025 (0.002, 0.049) 0.030
PLR Log Q4	0.013 (−0.010, 0.037) 0.271	0.024 (0.001, 0.047) 0.044	0.021 (−0.002, 0.045) 0.071
*P* for trend	.130	.021	.048

*Notes*: outcome variable: TeloMean, exposure variable: PLR quartiles. Non-adjusted model I: adjusted for none. Adjust model II: adjusted for sex, age, and race. Adjust model II: adjusted for sex, age, race, education level, marital status, smoking, alcohol consumption (both continuous and categorical), diabetes, hypertension, and body mass index (BMI).

PLR = platelet-to-lymphocyte ratio, TeloMean = mean telomere length.

In contrast, the results for the Q2 and Q4 groups were less consistent across different models. The Q2 group did not show significant differences from the Q1 group in any of the models, while the Q4 group displayed a near-significant trend in the fully adjusted model (β = 0.021, 95% CI: −0.002 to 0.045, *P* = .071), though this did not reach statistical significance. Additionally, the trend test showed a significant trend across PLR Log quartiles in both the minimally adjusted (*P* for trend = .021) and fully adjusted models (*P* for trend = .048).

### 3.3. Nonlinear analysis

**Figure 2.**
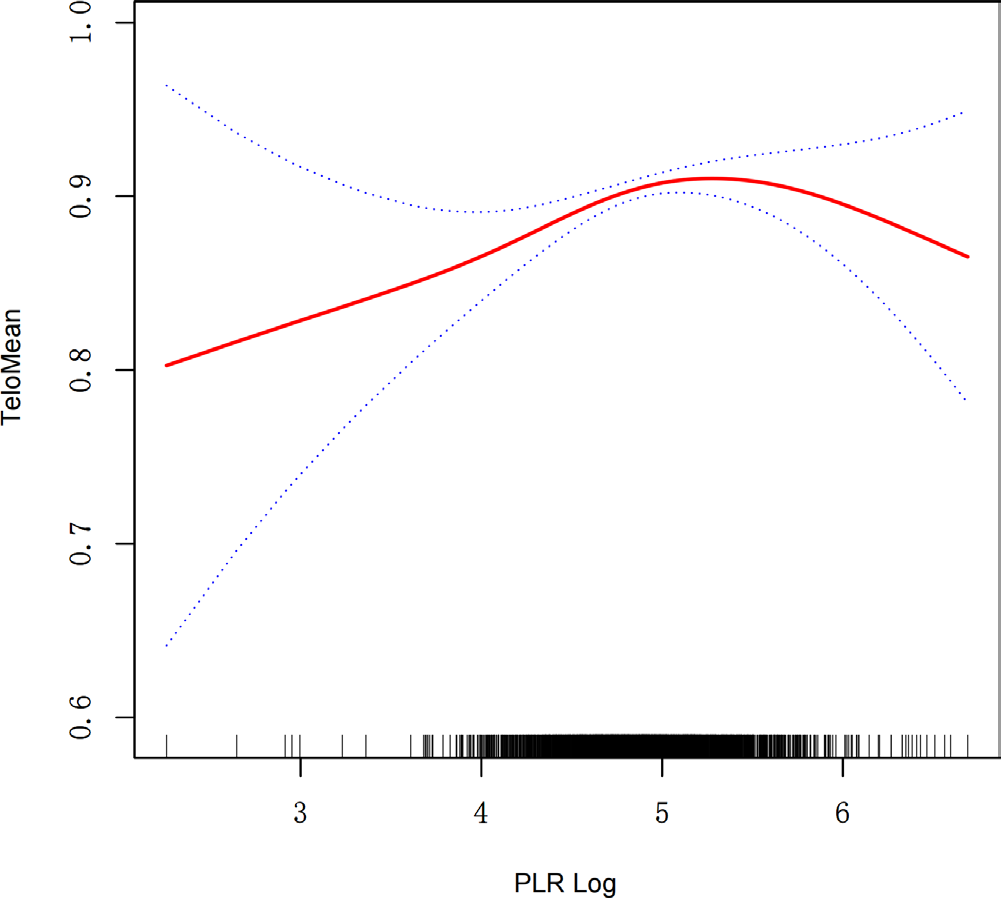
Smooth curve fitting for PLR Log and TeloMean. PLR = platelet-to-lymphocyte ratio, TeloMean = mean telomere length.

To investigate the nonlinear association between the PLR Log and TeloMean, we performed smooth curve fitting and segmented regression analysis (Fig. 2). After adjusting for all confounding factors, the smooth curve demonstrated an inverted U-shaped relationship between PLR Log and TeloMean (*P* = .035). The segmented regression analysis identified a threshold effect at PLR Log = 5.564 (Table 3). Below this breakpoint, TeloMean significantly increased with rising PLR Log (β1 = 0.034, 95% CI: 0.012–0.057, *P* = .003). However, when PLR Log exceeded the breakpoint, the association reversed, and TeloMean decreased significantly (β2 = −0.115, 95% CI: −0.221 to −0.009, *P* = .033). The difference in effect sizes between the 2 segments was significant (β2/β1 = −0.149, 95% CI: −0.264 to −0.035, *P* = .011).

**Table 3:** Threshold effect analysis of PLR Log and TeloMean.

PLR	TeloMean adjusted β (95% CI) *P* value
Model I	0.021 (0.001, 0.041) .040
Model II	
Breakpoint (*K*)	5.564
β1 (<*K*)	0.034 (0.012, 0.057) .003
β2 (>*K*)	−0.115 (−0.221, 0.009) .033
β2/β1	−0.149 (−0.264, −0.035) .011
Logarithmic likelihood ratio test *P*-value	.010

*Notes*: The model is adjusted for sex, age, race, education level, marital status, smoking, alcohol consumption, diabetes, hypertension, and body mass index (BMI).

PLR = platelet-to-lymphocyte ratio, TeloMean = mean telomere length.

The logarithmic likelihood ratio test (*P* = .010) further confirmed the presence of a threshold effect. These findings indicate that PLR Log is positively associated with TeloMean when below the breakpoint, but negatively associated when exceeding this value, supporting the hypothesis of a nonlinear relationship.

### 3.4. Subgroup analysis

To further evaluate the stability of the association between PLR Log and TeloMean across different demographic characteristics, we conducted a subgroup analysis. The results showed that age had a significant impact on the relationship between PLR Log and TeloMean (*P* < .05), indicating that this association varies across different age groups. However, no significant effect modification was observed in other stratified variables such as sex, race, smoking, alcohol consumption, diabetes, and hypertension (*P* > .05) (Table 4). This suggests that, apart from age, the negative association between PLR Log and TeloMean remains consistent across other demographic characteristics.

**Table 4:** Subgroups analyses of the effect of PLR Log on TeloMean.

Subgroups	N	β (95% CI), *P* value	*P* for interaction
Age			.005
60–65	775	0.015 (−0.026, 0.055) 0.483	
66–74	943	0.062 (0.029, 0.096) 0.000	
75–85	942	−0.011 (−0.041, 0.020) 0.493	
Sex			.585
Men	1355	0.026 (−0.001, 0.052) 0.058	
Women	1305	0.015 (−0.015, 0.044) 0.329	
Race			.364
Mexican American	552	0.032 (−0.014, 0.079) 0.171	
Other Hispanic	107	0.035 (−0.066, 0.135) 0.498	
Non-Hispanic White	1553	0.015 (−0.010, 0.040) 0.251	
Non-Hispanic Black	394	0.010 (−0.041, 0.061) 0.708	
Other race [including multiracial]	54	0.152 (0.016, 0.289) 0.029	
Smoking			.354
≥100 cigarettes in life	1413	0.029 (0.003, 0.054) 0.030	
<100 cigarettes in life	1247	0.010 (−0.021, 0.040) 0.528	
Alcohol			.709
<2 drinks/day	2072	0.019 (−0.003, 0.042) 0.096	
≥2 drinks/day	588	0.028 (−0.013, 0.069) 0.185	
Diabetes			.172
Yes	469	0.051 (0.003, 0.098) 0.037	
No	2191	0.015 (−0.007, 0.036) 0.190	
Hypertension			.236
Yes	1370	0.009 (−0.018, 0.037) 0.515	
No	1290	0.033 (0.005, 0.061) 0.023	

*Notes*: The model was adjusted for age, race, sex, education level, marital status, smoking, alcohol consumption, diabetes, hypertension, and BMI.

BMI = body mass index, PLR = platelet-to-lymphocyte ratio, TeloMean = mean telomere length.

## 4. Discussion

This study is the first to demonstrate an inverted U-shaped nonlinear relationship between PLR and TeloMean in older adults. Our findings indicated that TeloMean increased with log-transformed PLR within its moderate range before beginning to decrease as PLR Log increased further. Specifically, in participants in Q3 of this quartile, a significant positive association was observed (β = 0.025; 95% CI: 0.002–0.049, *P* = .030). In contrast, at the highest quartile (Q4), TeloMean slightly declined, though this decrease was not statistically significant (*P* = .071). Additionally, the trend test indicated a significant trend across PLR Log quartiles in the minimally adjusted (*P* for trend = .021) and fully adjusted models (*P* for trend = .048), further supporting the association. These results suggest that moderate levels of inflammation may help protect telomeres, while excessive inflammation could be detrimental.

Jurk et al identified chronic inflammation as one of the main drivers of telomere shortening.^[14]^ Over time, inflammation damages DNA directly through pro-inflammatory cytokines and oxidative stress exposure, accelerating cellular aging processes.^[15]^ Our findings align with Jurk et al’s work. We found that an increase in PLR Log may adversely impact TeloMean through these mechanisms, indicating an imbalance in pro-inflammatory and immune regulatory responses within the body. Liu et al found that PLR is linked to the prognosis of cardiovascular patients, where higher PLR values correlate to poorer outcomes.^[16]^ Kurtul et al also found that higher PLR values are linked to adverse cardiovascular outcomes.^[17]^ Studies have suggested that PLR Log measures both pro-inflammatory states and declines in systemic immune function, and our results add further support to this idea. Our data further demonstrate its value as an inflammatory marker, suggesting that an increase in PLR Log may speed telomere attrition rates among older adults, thus hastening their aging process.

Notably, our study revealed a striking inverted U-shaped relationship between PLR Log and TeloMean, which was especially evident among participants aged 66 to 74 (β = 0.062, 95% CI: 0.029–0.096, *P* < .001, *P* for interaction = .005). This phenomenon could be linked to age-associated immune decline and “inflammaging,” Franceschi et al have suggested that, with increasing age, chronic low-grade inflammation accumulates over time and leads to immune dysfunction as well as chronic diseases in elderly populations.^[18]^ Giunta et al further explored this theory and have observed similar findings.^[19]^

Our subgroup analysis further indicated that the association between PLR Log and TeloMean was significantly modified by age (*P* for interaction < .05), whereas no significant interactions were observed for other demographic factors such as sex, race, smoking, alcohol consumption, diabetes, and hypertension (*P* > .05 for all). These results suggest that elevated PLR Log may amplify age-related inflammation and contribute to telomere shortening, particularly in individuals aged 66 to 74.

Although our study revealed a strong association between PLR Log and TeloMean, this relationship did not reach statistical significance for those in the highest PLR Log range (Q4: β = 0.021, 95% CI: −0.002 to 0.045, *P* = .071). Under an increased inflammatory load, other biological mechanisms could mediate telomere shortening. Research by Zhou and Lee suggests that excessive inflammation responses may contribute to tumor formation and chronic diseases through various mechanisms, providing support for our speculation regarding high PLR Log’s exacerbating role in telomere attrition.^[20–22]^

Additionally, previous studies have found nonlinear associations between telomere length and various hematological parameters and cardiovascular diseases. For example, Dilixiati et al demonstrated an inverted *J*-curve relationship between leukocyte telomere length and erectile dysfunction, where shorter telomeres were associated with a higher risk of erectile dysfunction, but this association plateaued beyond a certain threshold.^[23]^ Similarly, Ainiwaer et al found that red cell distribution width and the red cell distribution width-to-platelet ratio were nonlinearly associated with cardiovascular diseases, suggesting that hematological parameters play an important role in disease progression through inflammatory mechanisms.^[24]^ These studies, along with our findings, highlight the potential role of hematological inflammation markers in aging-related diseases, further supporting the complex interplay between immune dysregulation, inflammation, and telomere attrition.

Future studies should investigate interactions between PLR Log, SII index markers, oxidative stress markers, and PLR Log to better understand their impact on telomere length and aging.^[25]^ Chronic inflammatory conditions, such as periodontitis, have also been linked to vascular calcification and other age-related diseases, further supporting the role of systemic inflammation in aging and disease progression.^[26]^ Additionally, recent studies have explored the associations between inflammatory and neurological biomarkers, such as serum neurofilament light chain, and chronic diseases, indicating their potential roles in systemic aging and disease progression.^[27]^

Strengths of this study include its use of a large NHANES sample, which enhances broad representativeness and applicability. We adjusted for multiple confounders to ensure robust findings. Nonetheless, there are some limitations to this research that must be addressed. First of all, as a cross-sectional study, we cannot establish causality between PLR Log and TeloMean. Longitudinal studies must be undertaken in order to further validate this nonlinear association. Second, although we adjusted for various confounds, residual confounders such as lifestyle factors or chronic diseases could still influence our results.

## 5. Conclusion

Our study identified an inverted U-shaped nonlinear relationship between PLR Log and telomere length, suggesting that moderate increases in PLR Log are linked to longer telomeres, while excessively high PLR Log levels may contribute to telomere shortening. The trend test confirmed a significant trend across PLR Log quartiles, and the threshold effect analysis further supported this nonlinear association. Our findings provide new insights into the complex relationships among inflammation, immune function, and telomere attrition in older adults, offering new directions for future research into PLR Log as a potential marker of aging.

## Author contributions

**Conceptualization:** Jiawei Ren, Delong Wang.

**Data curation:** Qinqin Liu.

**Formal analysis:** Qinqin Liu, Yue Gu, Yang Liu.

**Investigation:** Haixia Huang.

**Resources:** Haixia Huang, Delong Wang.

**Supervision:** Jiawei Ren, Delong Wang.

**Validation:** Yue Gu.

**Visualization:** Yue Gu.

**Writing – original draft:** Qinqin Liu.

**Writing – review & editing:** Delong Wang.
